# Ultra-shallow dopant profiles as in-situ electrodes in scanning probe microscopy

**DOI:** 10.1038/s41598-022-07551-3

**Published:** 2022-03-08

**Authors:** Alexander Kölker, Martin Wolf, Matthias Koch

**Affiliations:** Department of Physical Chemistry, Fritz Haber Institute of the Max Planck Society, Faradayweg 4-6, 14195 Berlin, Germany

**Keywords:** Chemistry, Materials science, Nanoscience and technology, Physics

## Abstract

The application of nano materials to control advanced functionality in semiconductor devices has reached the atomic scale. At this dimension the exact chemical and structural composition of a device is crucial for its performance. Rapid inspection techniques are required to find the optimal combination among numerous materials. However, to date the earliest electrical inspection is carried out after multiple fabrication processes. This delay makes the fabrication of atomically designed components very challenging. Here, we propose a sample system to chemically characterize nanoscale devices in-operando. We introduce ion-implanted contacts which embedded in the sample serve as additional electrodes to carry out scanning gate experiments. We demonstrate that the presence of these electrodes does not deteriorate the surface quality. The potential of this approach is highlighted by controlling the charge state of single dangling bonds on the silicon surface. Apart from our novel sample holder, the experimental setup was not modified making this approach compatible to most commercial low-temperature scanning probe microscopes. For silicon based devices, the versatility of this method is a promising avenue to gain a detailed and rapid understanding of functionalized atomic devices and quantum interactions at the atomic level.

## Introduction

In the last decade the insatiable demand for computational power has boosted substantial progress in semiconductor device fabrication by scaling components down to the smallest dimension. The next leap will be achieved by novel types of materials that allow advanced functionalization at the atomic level. This level of control should enable utilizing quantum mechanical effects for the next generation of atomic-scale devices that put exciting computational concepts based on quantum computation or spintronics in reach^1–5^. To blueprint the atomic structure of nano-scale devices it is crucial to understand the underlying physics. Only then their full potential can be unlocked. Although scanning probe microscopy (SPM), with its outstanding spatial resolution, is sensitive to both the structural composition as well as the electrical properties of the material, the experimental capabilities are limited by the vertical arrangement of the junction between tip and sample^6,7^ providing only one out-of-plane electrode to study complex nano scale devices. For this reason it is usually impossible to characterize nano-devices in operation with conventional scanning probe microscopes. These restrictions of the experimental setup have been addressed by multi-tip scanning tunneling microscopes demonstrating the immense experimental capabilities that arise from additional electrodes for the investigation of electronic, magnetic but also mechanical effects of nano-devices^8^. Four independent tips are used to unravel the contact resistance from the actual conductivity of the nano-object^9,10^ while the local potential landscape of the nano-device can be controlled by one of the probes^11^. However, customizing an existing SPM with additional tips requires severe and cost-intensive modifications of the experimental setup.

As an alternative to multiple SPM tips, using a sample system with electrodes just beneath the surface can be more favorable. From an experimental point of view this approach has two considerable advantages: first, the lateral sub-surface contacts do not conceal the nano-material allowing a precise probing of the electrode-material contact as well as the exact chemical composition of the junction by SPM due to the non-existing height of the electrodes. Second, in combination with nano-scale lithography^12^ these electrodes can be employed as interface for the placement of dopants^13–17^ or molecules^12,18^ with STM precision for the fabrication and in-situ investigation of novel nano-circuits^19^. This concept is illustrated for a single-molecule device in Fig. 1a. In other words this approach allows to not only position dopants or single molecules with highest precision but also to electrically inspect these STM patterned nano-circuits simultaneously. In case of single-molecule experiments^20^, the role of the molecule-electrode contact can be characterized which is known to strongly influence the properties of the junction^21^. The versatility of SPM can be used to probe the local density of states^22^, the local resistivity^23^ or the local contact potential difference^24^ of the nano-junction in-operando. Only very recently this level of control has been reported elsewhere^25^.

Here, we propose to enhance commercial scanning probe setups by upgrading a Si(001) sample system with multiple ion-implanted electrodes embedded just beneath the Si(001) surface. These implanted electrodes consist of highly packed antimony (Sb) patches that serve as conductive metallic leads. Solely the sample holder needs to be adapted, allowing a much easier and cost-effective introduction of additional electrodes. In this work, we demonstrate the applicability of ion-implanted dopant structures in silicon as a feasible way to upgrade a commercial Createc GmbH low-temperature SPM with additional electrodes. Our results show that the presence of these electrodes does not deteriorate the Si(100) surface quality as atomically flat areas are found on top of the patterned implants. A sharp transition from metallic to insulating is observed when moving the tip away from the highly doped regions. The versatility of this approach is demonstrated by controlling the charge state of dangling bonds (DBs) on the silicon surface. Apart from a specially designed sample holder, the experimental setup was not modified, making this approach compatible to most commercial low-temperature SPMs.

## Results

**Figure 1:**
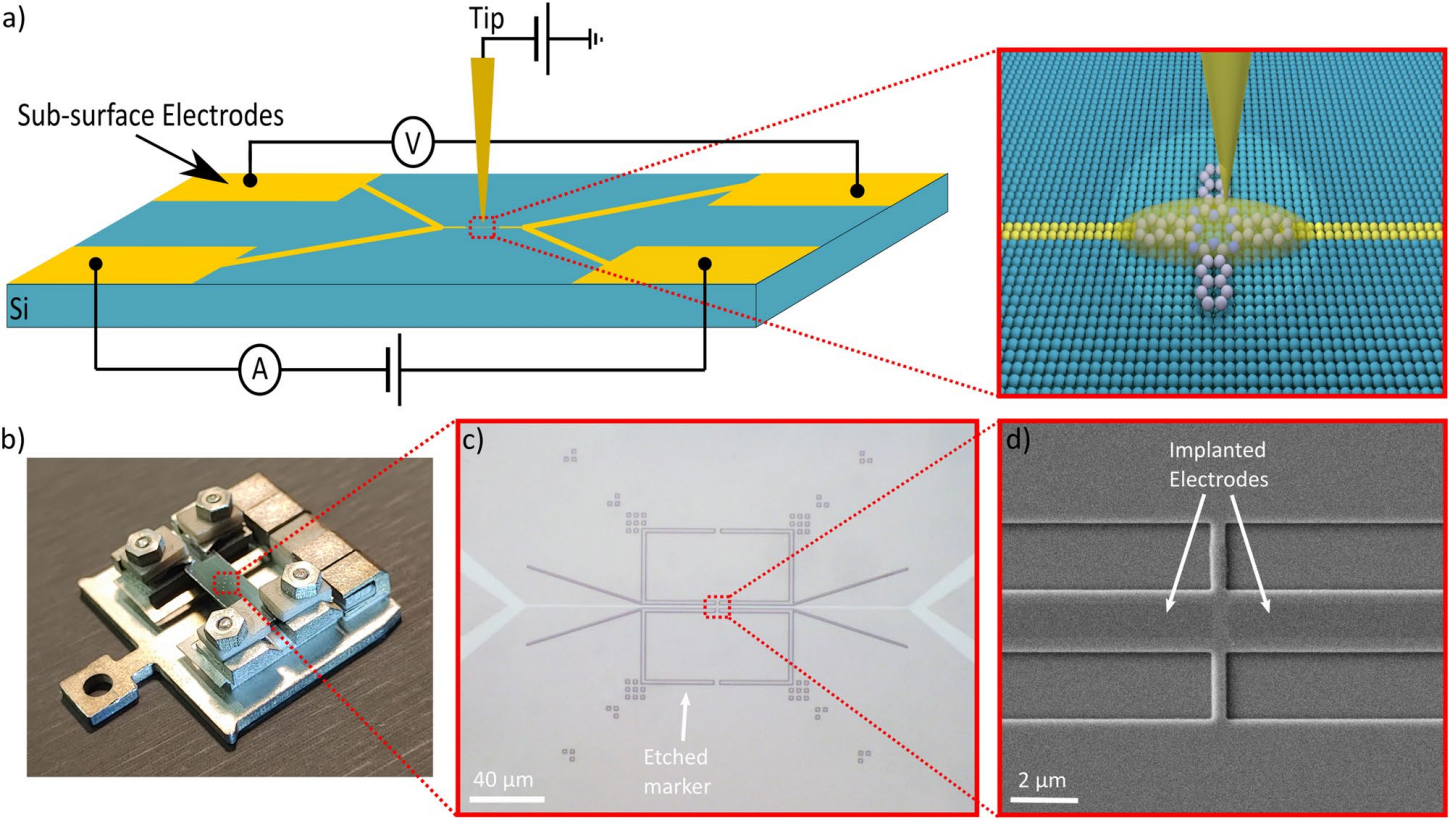
Illustration of the sample architecture and its concept for a STM precision fabrication and in-situ investigation interface of novel nano-circuits: (**a**) schematic illustration of the Si(100) sample system. Highly doped metallic regions are drawn in yellow, the silicon substrate is blue. The inset shows a possible single molecule experiment: with the aid of nano-scale lithography a molecule is placed with atomic precision between two narrow electrodes while the STM probe is utilized as in-situ investigation and gating tool. (**b**) Home-build sample holder for a Createc SPM system. (**c**) A microscope image of the sample comprising marker structure and integrated electrodes (white). (**d**) SEM image showing the deep etched central marker structure and two implanted electrodes (dark color) separated by a gap of 500 nm.

### Fabrication and design of the sample system

To electrically contact the implanted electrodes we have developed a home-build sample holder to independently read-out each ultra-shallow electrode (shown in Fig. 1b). The materials of the sample holder, $\hbox {AlO}_2$ and molybdenum, are chosen to survive high-temperature treatment such that the individual contacts and the sample itself remain electrically isolated from the sample plate. Four contact springs establish a reliable and solid contact to the macroscopic doped contact pads on the sample by pressing selectively against each sample corner where antimony has been implanted at a target depth of $\sim$ 10 nm with a density of $1\times 10^{-14}$ $\hbox {cm}^{-2}$ (yellow patches in Fig. 1a). For details about the ion implantation process the reader is referred to the methods section. Because of the low diffusion rate of antimony the ion-implanted structures are expected to withstand the high-temperature treatment necessary to prepare a clean and flat Si(001) surface^26^. More importantly these rapid-thermal anneal (RTA) steps promote Sb dopants to diffuse towards the surface^26^. As a result a good electrical contact between the sample holder clamping system and the electrodes is expected. Each sample is equipped with a dry-etched marker system (200 nm deep) allowing a convenient positioning of the STM probe in the marker center and finally to locate the $\mu$m large electrodes with the tip (see Fig. 1c,d)^27^. Note that the shape of the electrodes in the marker center can be tailored to match the specific requirements of the scientific question.

### Electrical inspection of the ultra-shallow electrodes

**Figure 2:**
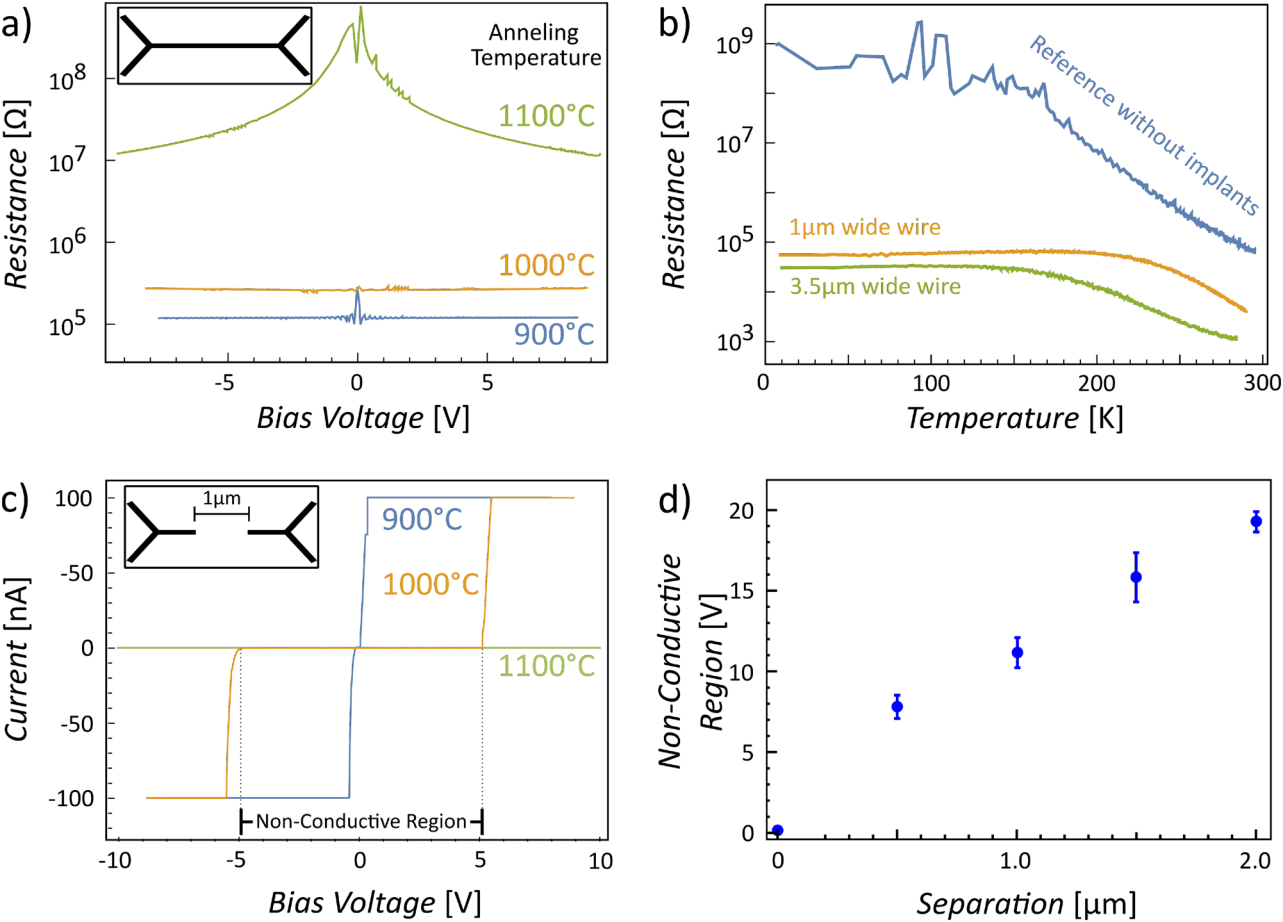
Electrical characterization of the ion-implanted regions: four probe resistance of  200 $\upmu$m long ion-implanted wires with and without gap. (**a**) Influence of the RTA temperature and (**b**) sample temperature on the resistance of the ion-implanted Sb-wire with varying width. The RTA temperature for **(b)** was 1000 $^{\circ }$C. The 0 V peak of the 900 $^{\circ }$C trace in (**a**) is a measurement artefact caused by the used current amplifier. (**c**) Non-conductive gap formation for RTA temperatures above 1000 $^{\circ }$C. (**d**) Non-conductive voltage region as a function of electrode separation demonstrating a linear relation.

First we investigate the electrical contact between the ultra-shallow electrodes and the sample holder as function of annealing temperature which is crucial for two reasons: first it is one of the most important parameters for the preparation of atomically clean and flat silicon surfaces (see methods section for further experimental details). Second, too high annealing temperatures lead to out-diffusion of the Sb dopants and harm the electrical integrity of the electrodes. Clean surfaces and ohmic electrodes, necessary for the correct electrical interpretation, are achieved only within a very limited temperature range. As a result the thermal budget for the surface preparation needs to be optimized, as any electrical contributions in the measurement signal which are caused by faulty, non-ohmic electrodes will jeopardize the analysis of such sophisticated experiments. The here proposed four-probe setup (see Fig. 1) allows us to selectively determine the resistance of a Sb-wire in the marker center, where a 200 $\mu$m long ion-implanted wire with a width of a 1 $\upmu$m has been implanted. To quantify the influence of the RTA temperature on the conductance of this wire, the device has been heated subsequently for 60 s to 900 $^{\circ }$C, 1000 $^{\circ }$C and 1100 $^{\circ }$C, respectively. The exceptionally high dopant density of the Sb ion-implanted regions is evident in the metallic character of the current signal even at 4 K. While the silicon host becomes insulating at low temperature, the current signal measured through the ultra-shallow electrodes still increases linearly with voltage, which is reflected in a constant resistance in Fig. 2a (blue curve).

Up to an annealing temperature of $\sim \, 1000\,^{\circ }$C a metallic character is confirmed (orange curve in Fig. 2a). Only when reaching 1100 $^{\circ }$C its resistance gradually increases for small bias voltages, which is an indication for dopant out-diffusion^26^ and/or significant surface depletion of Sb dopants similar to those found for As^28–30^. According to these results, the annealing temperature must remain below 1000 $^{\circ }$C to avoid damaging the electrical integrity of the ion-implanted Sb wires.

The dominant charge transport path through the Sb wires is determined via the help of a reference sample cut from the same wafer (1–20 $\Omega$cm) comprising no ion-implanted structures. Figure 2b compares the resistance of the reference sample to two 200 $\upmu$m long Sb wires of varying width (1 and 3.5 $\upmu$m) as a function of sample temperature. With decreasing temperature the low-doped silicon host becomes insulating. This is reflected in the measured resistance of the reference sample which increases continuously with lower temperature until exceeding the limit of the experimental setup (see blue curve in Fig. 2b). This is in stark contrast to the samples with ultra-shallow ion-implanted electrodes. Although the resistance increases at first when cooling the sample from room temperature to 200 K it plateaus at 10$^{5}$ $\Omega$ (see orange and green curve in Fig. 2b). In other words below $\sim$ 200 K the Sb ion-implanted wire dominates the charge transport. With decreasing wire width (3.5 $\upmu$m to $1\,\upmu \hbox {m}$) we observe only a small increase of $~24\%$ in resistance at low temperatures.

To estimate the contact resistance of our sample-holder clamping system we compare the four-probe resistance of the ion-implanted wires $R_{4T~wire}$ to the resistance measured in a two-probe geometry $R_{2T}$ at 10 K: the resistance increases approximately by 50 k$\Omega$. From the formula for the two terminal resistance $R_{2T}= R_{4T~wire}+ 2\cdot R_{contact}$ we conclude that each contact adds 25 k$\Omega$ of contact resistance to the overall resistance at low temperatures. This shows, that the sample-holder clamping mechanism forms a stable contact to our implanted electrodes.

### Ion-implanted two-probe contact

In future, the ion-implanted structures will be extended by atomic-scale STM dopant placement to realize nano-scale contacts as illustrated in the inset of Fig. 1a. Due to the reduced SPM scan-range at low temperature ($\sim 800$ nm) the distance between the ion-implanted electrodes needs to be minimized. At the same time this distance needs to be sufficiently large to prevent electrically shorting of the gap due to thermally activated dopant diffusion^31^.

To evaluate the influence of the RTA temperature on the conductance of two in-plane electrodes separated by a gap, we have first measured four-probe transport characteristics between two 1 $\upmu$m wide electrodes separated by a gap of 1 $\upmu$m (see Fig. 2c) for various RTA temperatures. After a 900 $^{\circ }$C anneal the electrodes display a strong metallic character. This changes when annealing the sample to 1000 $^{\circ }$C or higher, where a 10–12 V large symmetric insulating gap appears at low-bias voltages. The npn-device architecture yields a symmetric current trace where the first np-junction is operated in reverse for positive voltages, while the second pn-junction is in reverse mode for negative voltages. In addition to the already described four-probe wire measurement above, this observation further narrows down the optimal RTA temperature of our sample system. Below 1000 $^{\circ }$C the intrinsic/stray dopant concentration is too high and the formation of an electrically insulating gap is not observed for electrodes separated by 0.5 $\upmu$m. To conclude, RTA steps to 1000 $^{\circ }$C provide both electrical integrity of the Sb ion-implanted wires and an electrically symmetric insulating gap.

In Fig. 2d, we plot the width of the non-conductive zero-current region as a function of electrode separation. Here, we define the breakthrough voltage as current values larger than $3\times 10^{-9}$ A. Each sample has been annealed to 1000 $^{\circ }$C for 60 s before electrical characterization (Three 0.5-$\upmu$m, two 1-$\upmu$m, four 1.5-$\upmu$m and two 2-$\upmu$m samples). We observe a linear increase of the non-conductive region with increasing electrode separation, in unison with a linear growing depletion layer width extending further into the low p-doped substrate^32^.

### STM characterization of the electrodes

After a successful determination of the optimal RTA temperature for the preparation of electrically intact electrodes we use the STM capabilities to precisely characterize the electrode-gap transition region. A prerequisite to employ scanning probe microscopy to study nano-scale junctions are atomically flat and non-contaminated surfaces. Therefore, the surface quality at the interface between the undoped silicon substrate and the ion-implanted electrodes is of particular importance. We use our home-build sample holder to contact the implanted circuit in-situ and position the SPM tip on the gap-electrode interface with the help of deep-etched marker structures. This is easily possible even at 4 K when the scan range of the tip is limited to 800 nm $\times$ 800 nm.

**Figure 3:**
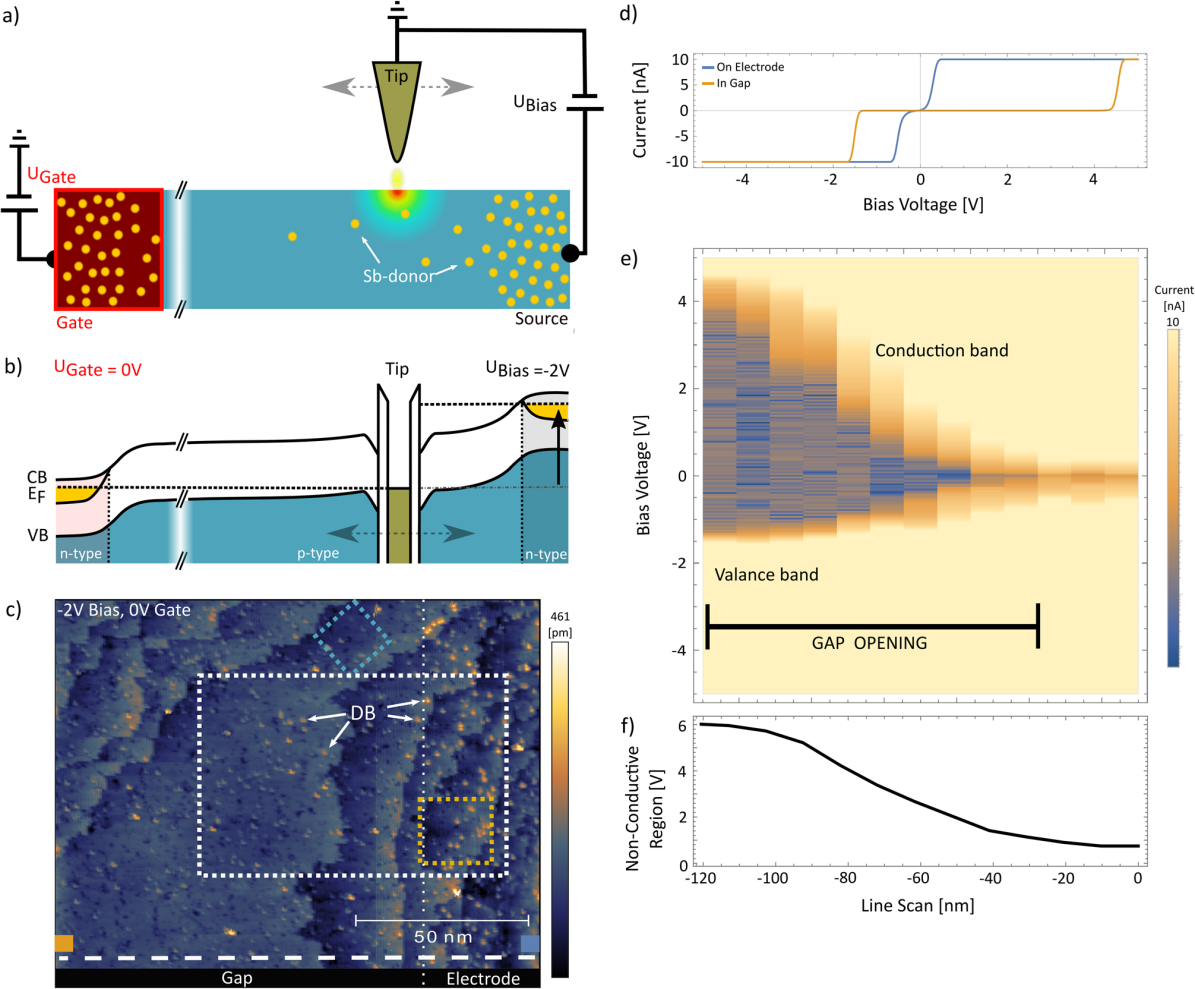
Electrical and topographic characteristics of tip-gap-electrode interface region without applied gate voltage: (**a**) schematic illustrating of the scanning tunneling spectroscopy method used to characterize the interface region. **(b)** Energy diagram of the npn-architecture at the Si-vacuum interface for an applied bias voltage of $U_{Bias}=-2~V$ pointing out transport characteristics. $E_{VB}$ and $E_{CB}$ are the valance band and conduction band edges, respectively, while $E_F$ displays the level of the Fermi energy. The impurity band of the highly doped electrode region (yellow) resides in the band gap of silicon. (**c**) STM filled state image of the interface region. The change of dangling bonds (DBs) appearance indicates the doped source electrode region. Areas demarcated with blue, orange and white rectangles correspond to the location where STM topography images are acquired at different bias and gate voltages shown in Figs. 4b–d and 5a–c, respectively. (**d**) Two IV curves acquired on the conductive source region (blue curve) and within the insulating gap (orange curve; set-point current 100 pA, set-point voltage − 5 V, $U_{Gate}=$ 0 V, z-offset − 1.5 nm). The location of the IVs are indicated by coloured squares in (**c**) accordingly. (**e**) Log-Current map with the tip in contact (− 1.5 nm offset) to the surface reveals a $\sim$ 100 nm transition from metallic to insulating at the electrode-gap interface. The spectroscopy data is acquired along the dashed line highlighted in (**c**). (**f**) Plot of the non-conductive region (extracted from current map in (**e**)) as a function of distance to the source electrode (current cut off 300 pA).

We prepare the sample according to our electrical findings by a RTA to 1000 $^{\circ }$C and perform a subsequent hydrogen termination following a standard procedure^33^. A STM topography filled state image acquired at $U_{Bias}= -2$ V at the electrode-interface region is depicted in Fig. 3c. It reveals step edge bunching at the electrode-gap interface with a slightly increased void defect density^34^ at the gap-electrode interface region. Large atomically flat terraces mask the transition from metallic electrodes to insulating Si(001) host. This proves that the presence of the electrodes does not deteriorate the surface quality. Even more importantly the electrodes are fully embedded in the surface in compliance with high-quality SPM experiments.

The high spatial resolution of SPM will be used to investigate the electrical properties of the electrodes, in particular the interface between the electrodes and the silicon host. A sharp transition from almost metallic to insulating is expected when traversing from the highly n-type doped Si (electrode) to the low-doped p-type Si host region. To probe the local conductivity scanning tunneling spectroscopy (STS) is conducted as illustrated in Fig. 3a. From now on, the electrode closer to the tip is called bias-electrode while the electrode further apart is referred to as gate-electrode. Furthermore, we want to emphasis that the SPM tip is set to ground and 0 V is applied to the gate electrode as the inspection is focusing on the electrical characteristics of the bias-electrode. Accordingly, the dominant transport path occurs via the bias-electrode to the SPM tip. When the SPM tip is located at the bias-electrode (see filled blue square in Fig. 3c) the IV trace displays a metallic character and electron transport occurs already at low bias voltages (blue curve in Fig. 3d). Next, the tip is placed in the gap region, $\sim 100$ nm away from the bias-electrode (see filled orange square in Fig. 3c). In contrast to before, the current path now traverses via a pn-junction as schematically depicted in Fig. 3a,b. Experimentally, a non-symmetric current trace is observed as depicted in Fig. 3d (orange curve). At negative bias voltages current transport occurs below − 1.5 V while for positive bias voltages (< 4 V) current is suppressed. A negative bias voltage drives the junction in forward mode (current flow is enhanced), while for positive bias voltages the junction is operated in reverse (current flow is suppressed).

To study this asymmetric non-conductive region further, we have performed a set of STM point spectroscopy (set-point current 100 pA, set-point voltage − 5 V, $U_{Gate}=$ 0 V, z-offset − 1.5 nm) as indicated by the white dashed line in Fig. 3c. Figure 3e shows the corresponding log-scale current map. An expanding non-conductive regions appears in the IV spectra when gradually moving the SPM tip further apart from the bias electrode (see also Fig. 3f) in which the non-conducting gap increases up to $\sim$ 6 V (current cut off 300 pA). The most obvious explanation for a rather smooth transition from metallic to insulating rather than a sharp cut off is the presence of out diffused donor or stray dopants in the p-type doped gap region that migrated from the implanted electrode regions during RTAs. In this case we expect the stray donor density to be dependent on the distance to the source electrode.

The energy diagram of our npn-architecture including the SPM tip is depicted in Fig. 3b. In equilibrium (left side of Fig. 3b) the Fermi energy level of the electrodes (highly doped n-type silicon) resides close to the conduction band (CB) level while for the Si host (low-doped p-type silicon) it locates close to the valence band (VB) level. In equilibrium both Fermi levels align resulting in an increased contact potential barrier formation between both regions, typical for a pn-junction. On the right side of Fig. 3b a low negative voltage is applied to the bias electrode. With increasing separation between tip and bias electrode, the required electrical field to bring the valance band in line with the work function of the tip increases. This explains the observed down shift of the current onset to lower bias energies in Fig. 3e. A local downward bending of the energy bands at the p-doped region underneath the probe is also considered in Fig. 3b. This tip-induced band-bending (TIBB) is caused by the local electric field beneath the SPM tip apex.

### Three-terminal controlled ionization of a dangling bond

**Figure 4:**
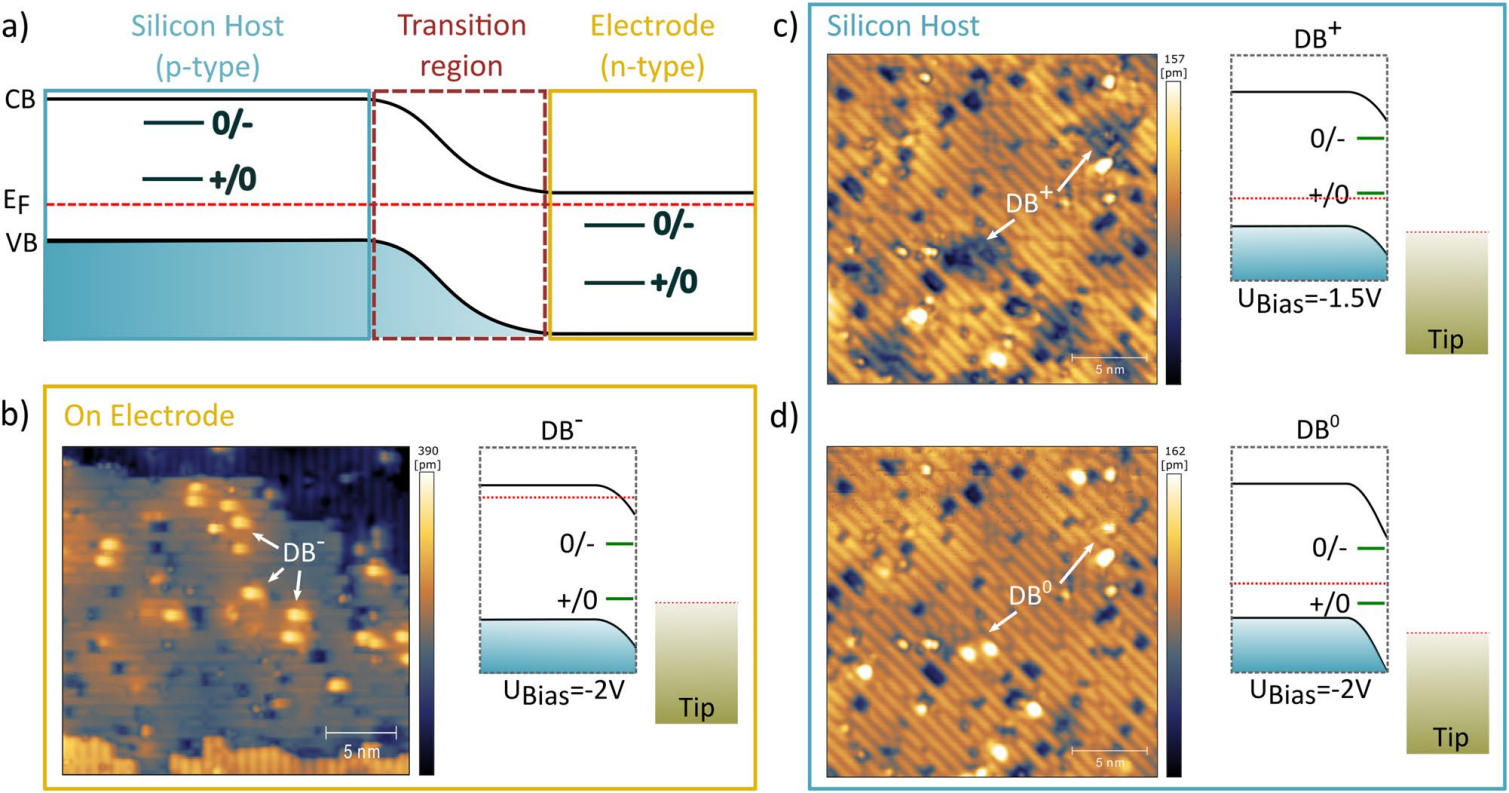
Controlling the charge state of a dangling bond: (**a**) schematic illustrating of the band structure depending on the position and the corresponding dopant concentration in the transition region. The position of the sample Fermi level with respect to the DB charge transition level determines their charge state. (**b**) STM image of the occupied states obtained on top of the electrodes (− 2 V) (position indicated as yellow rectangle in Fig. 3c) depicting a negatively charged DB. (**c**) STM image of the occupied states inside the gap region (position indicated as blue square in Fig. 3c) at − 1.5 V and (**d**) − 2 V, probing positive and neutral DB charge states, respectively. For each STM image the band structure and the effect of tip-induced band-banding is illustrated schematically.

We will now use the characteristic appearance of dangling bonds (DBs) on the Si:H surface for the electrical inspection of our implanted electrodes.

In total three charge states have been reported for DBs, i.e. $\hbox {DB}^{0}$, $\hbox {DB}^{-}$ and $\hbox {DB}^{+}$ with the corresponding two charge transition levels labeled +/0 and $0/-$^29,35^. The different appearances of DBs in STM i.e. the occupation of their charge states have been interpreted by Labidi and Rashidi et al. as rate dependent non-equilibrium tunneling governed by a voltage induced ionization of nearby subsurface dopants^29,30^. Complementary to this, Haider et al. observed that the charge state of dangling bonds is strongly influenced by the dopant concentration of the substrate^35–37^, which shifts the position of the Fermi-level $E_{F}$ with respect to the valence/conduction band (see also Fig. 4a)^38^. Accordingly, DBs are negatively charged on highly n-type doped H-terminated surfaces ($>10^{18}$ cm$^{-3}$), whereas appearing neutral for low n-type doped substrates^35–37^. On the other hand DBs are positively charged on p-type silicon^36,37,39^.

In our work we can distinguish these different charge states by STM imaging:

- $\hbox {DB}^{0}$: Appears as bright protrusion when probing the (un-)occupied states
- $\hbox {DB}^{-}$: Appears as bright protrusion when probing the occupied states and surrounded by a dark halo when probing the unoccupied states
- $\hbox {DB}^{+}$: Surrounded by a dark halo when probing the occupied states, but appears as bright protrusion when probing the unoccupied states

Our sample architecture exhibits regions of strongly varying dopant density. Therefore, we expect the charge state of the DBs to alter depending on their location (Fig. 4a). Imaging the gap region with low positive bias voltages (unoccupied states) is not possible due to the pn-character of the electrode-silicon host interface. Therefore, we focus on the DB appearance at negative bias in filled state images.

DBs on top of the electrodes should be negatively charged whereas DBs at the p-type silicon host are expected to be positively charged. This is in agreement with our experimental results. On top of the electrodes DBs appear negatively charged depicted as bright protrusion when measuring the occupied states (see Fig. 4b). In contrast, we observe positively charged DBs away from the electrodes for the low-doped p-type silicon host, indicated by the dark halo when imaging the occupied states (Fig. 4c).

At elevated negative bias voltages instead of a dark halo a bright protrusion appears in STM imaging (occupied states in Fig. 4d). Raising the bias voltage increases the effect of TIBB, until the +/0 transition of the DB is pulled below the Fermi-level of the sample. Accordingly the DB will be charged neutrally when reaching this threshold voltage and the dark halo assigned to a local positive charge disappears. This clearly indicates a neutral charge state when probing the occupied states.

The precise position of the +/0 transition for each DB depends strongly on its location but also on its environment such as other DBs, charge defects but also sub-surface dopants^29,30,40^. Therefore, we expect the threshold voltage to slightly differ for each DB which will be demonstrated in the following section.

### Gate influence on surface feature at the gap-electrode interface

To demonstrate the potential of the here proposed subsurface electrodes integrated into the sample architecture, we now use the second electrode to control the charge state of single dangling bonds. Here, the second electrode separated by $\sim 1.5~\upmu$m from the source is utilized to apply an additional voltage with respect to the tip. The applied voltages on either electrode is used to control the potential landscape in the gap regions which is directly reflected in the appearance of surface features, in this case DBs.

**Figure 5:**
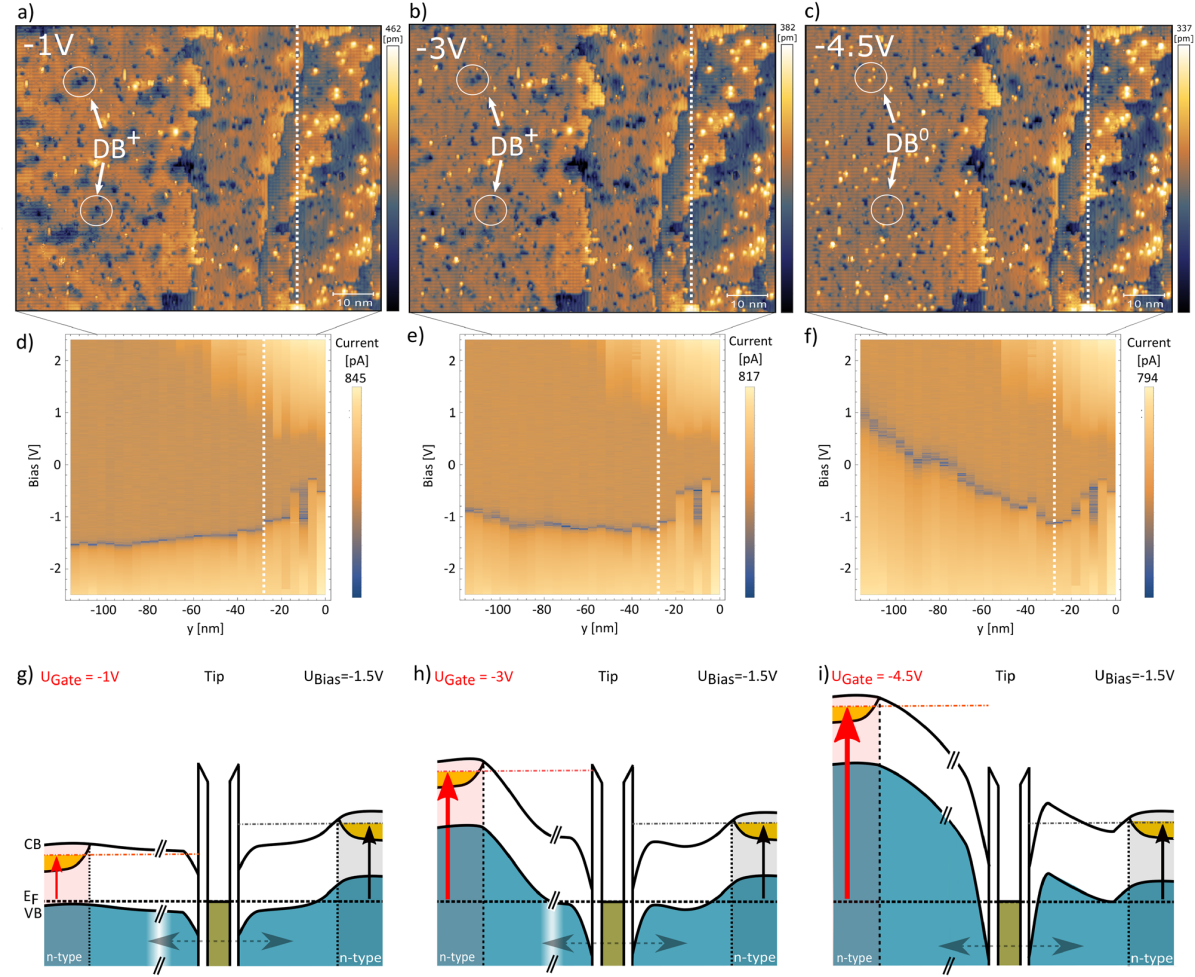
Characteristics and gate influence on surface feature at the tip-gap-source interface with applied gate bias: (**a–c**) individual STM topography images taken at different gate voltages (Fixed $U_{Bias} = -$ 1.5 V, 100 pA) and their corresponding Log-current plots (**d–f)**) (as acquired along the white dashed line indicated in Fig. 3b). Two white circles point out DBs in the gap region that change their appearance from depression to protrusion with increasing gate voltage. Energy diagrams (**g–i**) of the whole gate-gap-source-tip system for different gate voltages.

To study this effect STM images of the interface region for different applied gate voltages have been acquired at a fixed bias voltage of − 1.5 V (100 pA) as shown in Fig. 5a–c. The dominant features that change their appearance during this gate voltage sweep (white circles in Fig. 5a–c) are positively identified as DBs that change their appearance in the gap region from depression to protrusion (71.4 $\%$) with increasing gate voltage till reaching the metallic part of the electrodes (for more details see section S1 and S2 in the Supplementary Information). The white lines in Fig. 5a–f indicate the onset of the source electrode at $x = -28$ nm. Here the DBs appear as bright protrusion and the band gap remains unperturbed independent of gate voltage observed by STS current log maps shown in Fig. 5d–f. While DBs on the electrode region stay negatively charged throughout gate voltage changes DBs in the gap region undergo charge transition from positively to neutrally charged. As mentioned earlier the DB charge transition energy is strongly influenced by its local electrostatic environment on the surface, thus DBs with the same distance to source can display slightly different transition points as function of gate voltage. That explains the non-uniform DB appearance at different gate voltages at the same distance to source.

Figure 5d–f shows the influence of the gate voltage on the potential landscape at the bias electrode interface. Figure 5g–i depicts the corresponding energy diagrams of the whole gate-gap-source-tip system. We find that low gate voltages (− 1 V) have little effect on the position of the current onset at negative bias voltages. However, increasing the gate-voltage (− 3 V) results in an upward bending of the VB as a function of tip bias-electrode separation. This highlights that the impact of the gate-voltage on the position of the current onset increases with distance, which becomes especially obvious at − 4.5 V (Fig. 5f). Here, the VB is shifted above the tip Fermi-level, and the dominant transport channel now is via the gate electrode as indicated in the energy diagrams in Fig. 5g–i (Also see section S3 in the Supplementary Information for more details). Accordingly, the dominant current path is set by both the ratio of gate/bias-voltage as well as the tip position.

Finally, we want to address the difference between the two electrodes. From a technical point of view both electrodes should be identical. Highly n-type doped regions are embedded in the silicon host. The respective tip-electrode separation, results in different lever-arms, which sum up to a total electric field below the tip. The local total electric field strength under the tip governs the current onset position of the VB and also the position of the DB charge state transition and hence its appearance induced by TIBB. The electric field strength consists of a bias and gate voltage component and depending on the location of the tip between both electrodes the voltage coupling to the total electric field varies for each reservoir. Hence, depending on the tip position and the applied voltage at both electrodes the dominating current path can change. This becomes obvious when conducting bias spectroscopy at elevated gate voltages. Even for relatively large positive bias voltages a negative current is detected, which flows between the second gate electrode and STM tip (ground). Therefore, the gate electrode cannot be considered as a pure gate. Instead the complete npn-architecture needs to be considered to explain the experimental results. This is in contrast to phosphorous doped devices^14–16,27^ where the already mentioned symmetric device architecture is key. Each n-doped structure works as a gate, as long as the voltages are below the breakdown voltage of the npn-transistor. Only when exceeding the threshold voltage, a current starts to flow and the gating effectiveness decays. In our case, the situation is different. The tip operates predominantly in the gap region, where the npn-symmetry is non-existing. Therefore, a current flow from both electrodes occurs as soon as the voltage is larger then the much smaller breakdown voltage of the np-transistor.

## Conclusion and discussion

In summary, we demonstrated how to implement multiple additional electrodes into a commercial SPM system by just slightly modifying silicon sample and sample holder. In particular we validated how this sample system can be used to study charge transport of nano-scale devices in-operando, while using the tip and electrodes to precisely control the electrical potential landscape. This is possible because the presence of the ultra-shallow Sb doped electrodes does not affect the quality of the silicon surface. We found that electrodes featuring a minimum gap size of 500 nm are still displaying a non-conductive gap region of $\sim$ 8 V suitable for transport inspection experiments. From STS measurements we estimate a full gap opening to occur at $\sim$ 100 nm distance apart from each electrode, leading to a minimum electrode separation of 200 nm. Although samples comprising smaller gaps have not been examined in this study we thus believe that slightly smaller and non-leaking gap sizes could be realized following our method and preparation recipe. Finally, we demonstrated the veracity of the electrodes by successfully controlling the charge state of individual dangling bonds on the silicon surface. In future experiments the layout of the electrodes will be optimized to study true atomic-scale circuits. Thereby, scanning tunneling hydrogen resist lithography will be used for the fabrication of atomic-scale electrodes^16,25,41^ and the controlled placement of individual dopants and organic compounds^12,18^. Using this technique conducting areas can be patterned with sub nm-precision at low temperature in an otherwise insulating Si(100) host. Despite the experimental challenges which need to be resolved first, in future it might be possible to correlate the transport signal to local chemical variations (e.g. defects) and electronic properties (e.g. molecular orbitals). Electrical but also magnetic effects can be studied with atomic resolution and unprecedented control of the potential landscape and chemical composition of the junction. Nano-materials or nano-devices will be characterized in-operando to directly link the transport measurements to the precise chemical composition of the junction, resolved by STM/AFM with sub-molecular resolution^42^. The successful implementation and validation of additional in-plane electrodes into a commercial SPM system as reported in this study, will aid to understand the impact of the precise chemical composition of a junction on the device performance and paves the way towards truly atomisticaly engineered quantum-devices.

## Methods

### Scanning probe microscopy and sample preparation

A commercial low-temperature SPM from CreaTec Fischer & Co. GmbH is used with bias applied to the one electrode while tip and the remaining second electrode is on ground. All data were acquired at a sample temperature of 4.8 K if not stated otherwise.

The fabrication of the locally implanted electrodes was carried out at the Ferdinand-Braun-Institute in Berlin as an external commercial service. 200 nm deep marker structures were dry-etched into a 3-inch Si(100) wafer using reactive ion etching. Ultra shallow antimony dopant profiles were ion-implanted by a commercial medium current implantation system operating at 10 kV by utilising a ZEP 520 A resist as mask (200 RPM at 180 $^{\circ }$C) and MIBK as developer. Simulations indicate a peak concentration of implanted ions of approximately $1\times 10^{21}$ atoms/$\mathrm {cm^3}$ at a mean depth of approximately 10 nm away from the surface. In order to obtain macroscopic clean waver and junctions, free of any contamination, the wafer has been treated after ion implantation by multiple chemical cleaning steps including Sulphuric Peroxide, RCA2, hydrofluoric acid, acetone and isopropanol rinse before loading into ultra high vacuum (UHV). $3\times 10~\mathrm {mm^2}$ samples featuring ion implanted and deep etched structures are cleaved from the 0.5 mm thick Si(100) wafer exhibiting an resistivity of 1–10 Ohm/cm. An atomically flat silicon surface is obtained following a standard procedure^33^ consisting of a thorough outgas anneal of the samples overnight and a subsequent cycle of RTA steps to > 900 $^{\circ }$C monitored by a infrared pyrometer (PYROSPOT DT 40C) with an estimated uncertainty of $\pm 20\,^{\circ }$C. Subsequently the surface is passivated with a single layer of hydrogen.

For a detailed electrical inspection of two adjacent electrodes via transport measurements two $\sim$ 100 $\upmu$m long wires with a width of 1 $\upmu$m have been implanted (see white structures in Fig. 1c) that reach into the marker center (dark areas in Fig. 1d surrounded by the dry etched marker). Using this design multiple samples have been fabricated featuring a range of different electrode widths (1–3.5 $\upmu$m), no gap (wire) and a variety of gap sizes (0.5–2 $\upmu$m).

## Supplementary Information

Supplementary Information.
