# Supplementary material for: Ultra-shallow dopant profiles as in-situ electrodes in scanning probe microscopy
Source: Sci Rep. 2022 Mar 8;12:3783. doi: 10.1038/s41598-022-07551-3 (PMC8904578; doi:10.1038/s41598-022-07551-3)
Supplement: Supplementary file 1 — Supplementary Information. [file 41598_2022_7551_MOESM1_ESM.pdf]

Supporting Information: Ultra-Shallow Dopant Profiles as In-Situ Electrodes in Scanning Probe Microscopy

Alexander Kölker[1,*], Martin Wolf[1], and Matthias Koch[1,*]

[1]Fritz Haber Institute of the Max Planck Society, Department of Physical Chemistry, Faradayweg 4-6, 14195 Berlin, Germany,
[*]koelker@fhi-berlin.mpg.de, m.koch@fhi-berlin.mpg.de

S1: Dangling bond charge transition

We can identify dangling bonds (DBs) close to the interface region and their charge states transition point from their appearance in STM images at different applied gate voltages. Figure S1 shows STM images of the interface region for -1 V (top) and -6 V (bottom) gate voltages acquired at a fixed bias voltage of -1.5 V (100 pA).

We identify 78 features that change their appearance after the gate voltage sweep. Four main distinguishable type of features are observed:

- A) Spherical depression that changes to protrusion labeled as x, (56) equals 71.8% (DBs),
- B) Depression that changes to flat surface labeled 0, (12) equals 15.4% (probably charged defects or Pb center),
- C) A strong protrusion that is changing into flat surface labeled with a large white circle, (8) equals 10.3% (probably subsurface donor) and
- D) the creation of a protrusion labeled as C, (2) equals 2.6% (probably DB creation).

We attribute the dominate feature A to be DBs because of their uniform appearance and spectral characteristics as shown in Figure S2.

STM filled state images and STS obtained on a DB for varying bias voltages reveal a localised state at -1.83 V (no gate voltage applied) in line to those found for DBs in literature[1]. The charge state transition voltage is strongly determined by the local electrostatic environment on the surface as mentioned in the main text and varies individually for each DB as shown in Figure S2d. While one DB (green arrow) undergoes charge transition at -4 V gate voltages, nearby DBs (red arrows) exhibit a slightly higher charge state transition voltage at -5 V gate voltage.

S2: Dominant charge transport path

Figure S3 demonstrates the impact of bias and gate voltage application on the current flowing to the tip. For a fixed tip position in the gap close to the source electrode and a constant bias voltage of -3 V an increasing gate voltage first displays only a small effect on the absolute current increase as shown in Figure S3b. For this particular tip-sample settings the current slightly decreases such that the tip is engaging the surface to maintain a constant current setpoint, see Figure S3a. When reaching a critical gate threshold voltage ($\sim$-3.5 V) electron transport via the gate electrode takes over as dominating charge transport path. The tip is retracted with increasing negative gate voltage to maintain a constant current set point.

References

1. Labidi, H. *et al.* Scanning tunneling spectroscopy reveals a silicon dangling bond charge state transition. *New J. Phys.* **17**, 073023, DOI: 10.1088/1367-2630/17/7/073023 (2015).

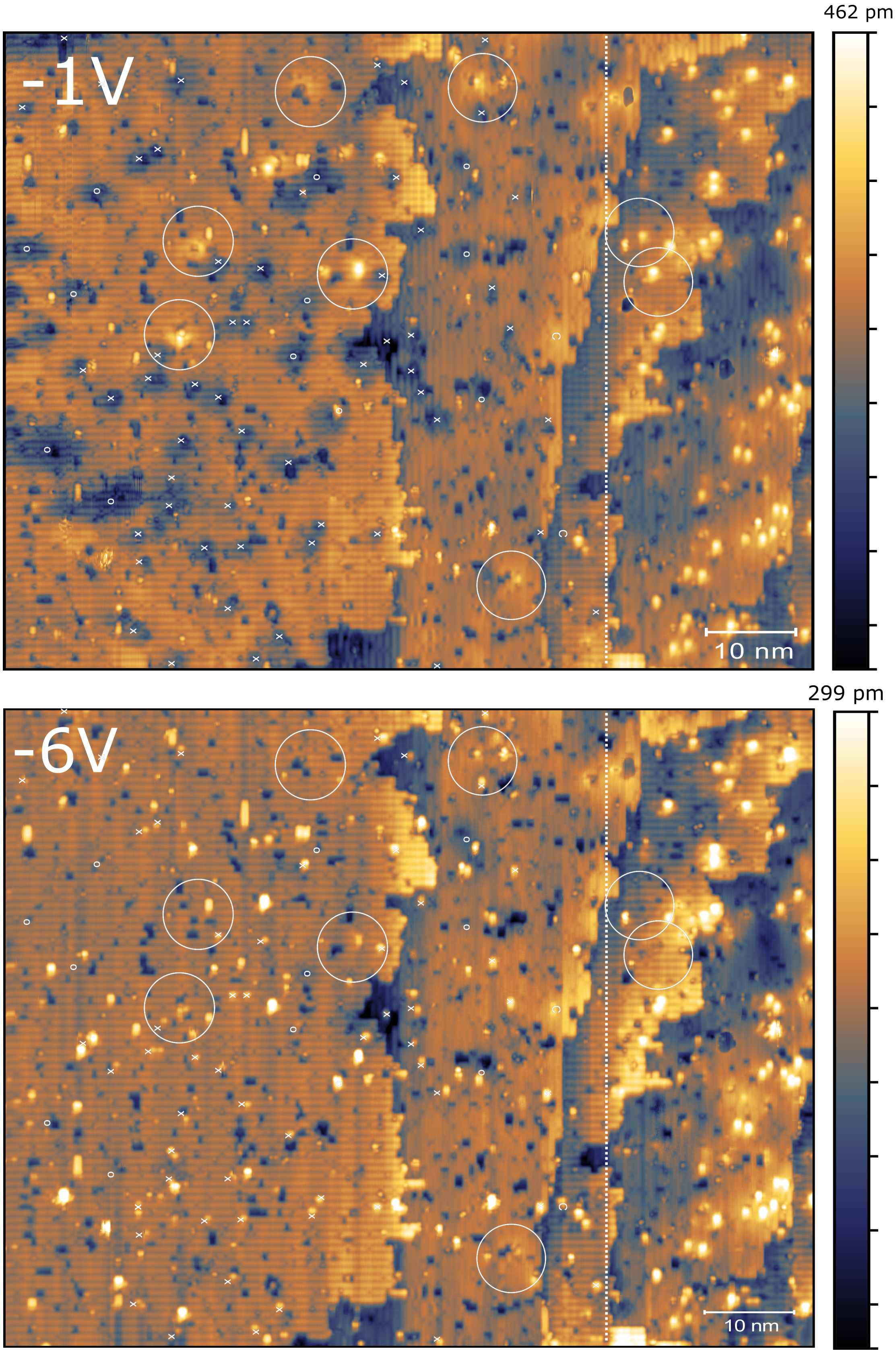

Figure S1. STM images of the interface region for -1 V (top) and -6 V (bottom) gate voltages acquired at a fixed bias voltage of -1.5 V (100 pA). The location is highlighted as white dashed square in Figure 3b. A total of 78 features change their appearance during gate voltage sweep four different type of features: A) Depression to protrusion (x,56), 71.8%, B) Depression to flat surface (0,12), 15.4%, C) Protrusion to flat surface (large white circle,8), 10.3% and D) DB creation (C,2) , 2.6%.

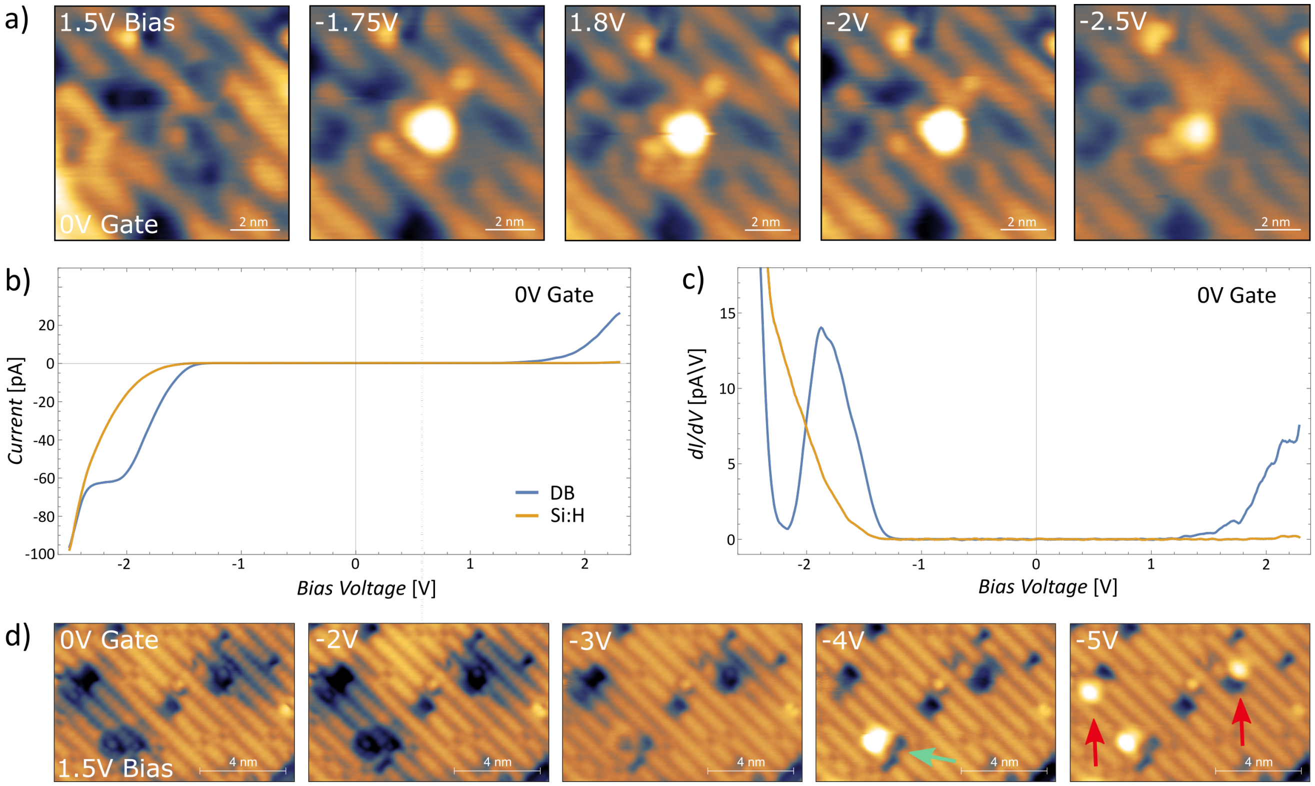

Figure S2. (**a**) STM filled state images for different bias voltages obtained close to the source electrode (15 nm to electrode) with no gate voltage applied. (**b+c**) STS obtained on terminated silicon and a DB reveals a localised state at -1.83 V. (**d**) Local influence of the gate on the charge transition voltage of DBs 41.5 nm away from the source electrode.

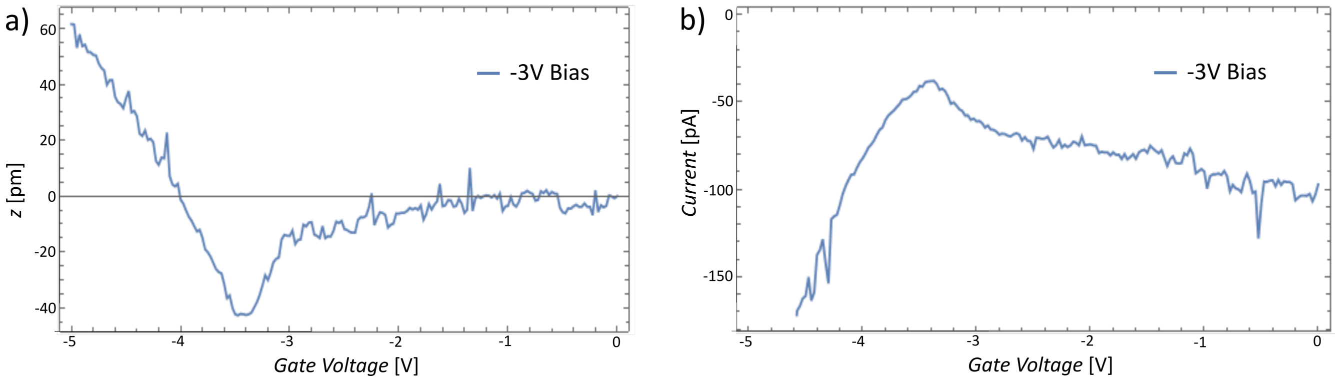

Figure S3. (**a**) Gate voltage plot against relative tip movement and (**b**) absolute current for an other sample (that is shown in Figure 3-5) and location close to the source electrode at -3 V bias. Till $\sim$-3.5 V gate voltage the dominant current transport occurs via the source electrode. With increasing gate voltage the current decreases (at a fixed z). On the other hand to maintain a constant 100 pA the tip is approaching the surface. Above -3.5 V electrons from the gate electrode dominate the transport and current increases (the tip is retracted).
